# Tex13a Optimizes Sperm Motility *via* Its Potential Roles in mRNA Turnover

**DOI:** 10.3389/fcell.2021.761627

**Published:** 2021-10-18

**Authors:** Yinchuan Li, Panpan Mi, Xue Chen, Jiabao Wu, Xiaohua Liu, Yunge Tang, Jinmei Cheng, Yingying Huang, Weibing Qin, C. Yan Cheng, Fei Sun

**Affiliations:** ^1^Institute of Reproductive Medicine, Medical School of Nantong University, Nantong, China; ^2^NHC Key Laboratory of Male Reproduction and Genetics, Guangdong Provincial Reproductive Science Institute (Guangdong Provincial Fertility Hospital), Guangzhou, China; ^3^The Mary M. Wohlford Laboratory for Male Contraceptive Research, Center for Biomedical Research, Population Council, New York, NY, United States

**Keywords:** Tex13a, CCR4–NOT, Cnot1, spermiogenesis, sperm motility, knock-out

## Abstract

mRNAs have been found to undergo substantial selective degradation during the late stages of spermiogenesis. However, the mechanisms regulating this biological process are unknown. In this report, we have identified *Tex13a*, a spermatid-specific gene that interacts with the CCR4–NOT complex and is implicated in the targeted degradation of mRNAs encoding particular structural components of sperm. Deletion of *Tex13a* led to a delayed decay of these mRNAs, lowered the levels of house-keeping genes, and ultimately lowered several key parameters associated with the control of sperm motility, such as the path velocity (VAP, average path velocity), track speed (VCL, velocity curvilinear), and rapid progression.

## Introduction

Dozens of genes have been reported to be transcribed enormously in round spermatids during spermiogenesis due to nucleus condensation and transcription termination, for later use during the late steps of elongating spermatids (ESs) (Kleene, 1989, 1996; Iguchi et al., 2006). Short truncated 3′-UTR, alternative splicing, changes in the site of polyadenylation, and increased rates of transcription in round spermatids for delayed translation have been adopted potentially to enhance the half-time of these mRNAs (Braun, 1998; Walker et al., 1999; Steger, 2001; Kleene, 2003; Liu et al., 2007; Cullinane et al., 2015; Li et al., 2016). However, it is unclear how these mRNAs are degraded once they have completed their roles and turned into junk RNAs during late ESs. The majority of published papers, on the other hand, relate to piRNA, the chromatoid body, and nonsense-mediated mRNA decay (NMD) in round spermatids (Meikar et al., 2011; Peruquetti, 2015; Jones and Wilkinson, 2017; MacDonald and Grozdanov, 2017). piRNAs have also been reported to play an important part in massive mRNA decay in the late spermatids (Gou et al., 2015). Similarly, CCR4–NOT was also reported to participate in the mRNA degradation in the late spermatids. For example, Cnot7, a nuclease of the CCR4–NOT complex, was knocked out in testis, resulting in significant germ cell defects and male infertility (Berthet et al., 2004). The CCR4–NOT complex is a multifunctional machinery that contains two different types of deadenylase enzymes that effectively shorten mRNA poly(A) tails (Bartlam and Yamamoto, 2010; Ukleja et al., 2016). However, the possible roles of the CCR4–NOT complex in modulating ESs largely remain unknown.

Three types of nucleases and multiple signaling pathways are involved in the breakdown of RNA (Garneau et al., 2007; Houseley and Tollervey, 2009; Arraiano et al., 2013). These nucleases and pathways usually play redundant roles in the cells. Among these pathways, the CCR4–NOT complex can effectively degrade poly(A) tails of mRNAs and initiate the early as well as the rate-limiting step of mRNA turnover (Wahle and Winkler, 2013). The CCR4–NOT complex, as a core and non-specific scissor for poly(A) tail, normally requires the help of other special partners, such as 3′-UTR regulatory elements and a variety of *trans-*acting factors, to trigger targeted decay of specific mRNAs (Mayya and Duchaine, 2019; Chalabi Hagkarim and Grand, 2020). Cnot1 is the largest subunit of the core CCR4–NOT complex and provides the backbone for other subunits to associate together (Temme et al., 2014; Chalabi Hagkarim and Grand, 2020). DND1 has been shown to bind Cnot1 and mediate the degradation of specific mRNAs in spermatogonia (Yamaji et al., 2017).

In this report, we have identified Tex13a, a spermatid-specific partner of CCR4–NOT complex, that can potentially bind to Cnot1 and alter the degradation of particular mRNAs encoding structural components of late ESs. Tex13a was initially revealed to repress transcription (Kwon et al., 2014, 2016; Kim et al., 2021). *Tex13* family is composed of four different members: *Tex13a*, *Tex13b*, *Tex13c1*, and *Tex13d* being transcribed specifically or predominantly in male mouse germ cells (Bellil et al., 2021; Kim et al., 2021). Tex13b was found to be highly expressed in spermatogonia, and Tex13d was found to be highly expressed in meiotic phase spermatocytes. Tex13a and Tex13c were found to be highly expressed in post-meiotic spermatids, which were ideal for exploring their roles in spermiogenesis. The C-terminal zf-RanBP2 domain of Tex13a can bind a consensus AGGUAA of single-stranded RNA (Nguyen et al., 2011). In this report, *Tex13a* was deleted and single-cell RNA sequencing (scRNA-seq) was adopted to monitor the transcriptomic changes during the late steps of ESs. Additionally, the phenotypes of *Tex13a* deletion and the changes of sperm motility were also evaluated in both Tex13a-KO and WT male mice.

## Materials and Methods

### Mice and Tex13a-KO Models

Male C57BL/6N mice (10–12 weeks old) were used for this study and animals were maintained under standard conditions. All animal protocols and experiments were approved by the University of Nantong Animal Care and Use Committee and the Animal Care and Use Office. Tex13a-KO C57BL/6N mice models were developed by Shanghai Model Organisms with CRISPR/Cas9. All the reading frames of Tex13a were deleted.

### Single-Cell Library Preparation and scRNA-Seq Data Analysis

Three unilaterally decapsulated testes from three mice from either Tex13a-KO or Tex13a-WT groups were pooled. The cell suspension preparation, cell capture, and library preparation were carried out according to previous reports (Dura et al., 2019; Li et al., 2021). scRNA-seq libraries were prepared by following the protocol of GEXSCOPE^TM^ Single-Cell RNA Library Kit (Singleron Biotechnologies, Nanjing, China). Briefly, mixed single-cell suspensions were loaded onto the microfluidic devices. Barcode beads were loaded onto the microfluidic device after the cells had settled into the wells. The cells were incubated with the lysis buffer for 20 min to facilitate the release of mRNA, which were then captured by the barcode beads. The beads were washed with 6× SSC twice after retrieval, and the captured mRNA was reverse-transcribed. The beads coated with cDNA were then amplified by PCR. The amplified DNA was purified followed by assessing its quality by Agilent BioAnalyzer using high-sensitivity chip. The purified cDNA was then pooled and used for the standard Nextera tagmentation and amplification reactions (Nextera XT, Illumina) using a custom primer instead of an i5 index primer to amplify only those specific fragments that contained the cell barcodes and UMIs. The libraries were sequenced on the Illumina HiSeq X using 150-bp paired-end reads.

The raw data analysis pipeline was conducted essentially according to our previous report (Li et al., 2021). Briefly, the raw data were processed by FastQC (V0.11.7) for quality evaluation, whereas fastp (v1) was used for trimming and STAR aligner (v2.5.3a) was used for alignment, while feature Counts (v1.6.2) were used for transcript counting. The cell barcodes and UMIs of the transcripts without polyT tails were extracted after they were filtered out. Adapters and polyA tails were thereafter trimmed before the second read was mapped to the UCSC mm10 reference genome with ensemble version 92 gene annotation. The reads with the same cell barcode, UMIs, and genes were grouped to calculate the number of UMIs generated per gene per cell. The cells from Tex13a-KO and WT testes were integrated and filtered under Seurat 3.2.3 (Stuart et al., 2019). The batch effects were thereafter removed by sctransform 0.3.1 embedded in the Seurat package. All the cell clusters were divided in an unbiased manner.

To identify the differentially expressed genes, the function FindMarkers of Seurat was used (test.use = “bimod,” logfc.threshold = 0.1). Upregulated genes (*p* ≤ 0.05, *p*_val_adj ≤ 0.05, logFC ≥ 0.2, and PCT1 ≥ 0.2) and downregulated genes (*p* ≤ 0.05, *p*_val_adj ≤ 0.05, logFC ≤ −0.2, and PCT2 ≥ 0.2) were filtered as the differentially expressed genes between Tex13a-KO and WT.

The gene set enrichment was performed following the competitive gene set enrichment test CAMERA embedded in the SingleSeqGset (version 0.1.2.9000) R package (Wu and Smyth, 2012; Cillo et al., 2020). mRNA surveillance pathway, deadenylation-dependent mRNA decay, and RNA degradation were derived from PathCards of GeneCards (Belinky et al., 2015). All other gene sets or pathways used were obtained from C5 ontology gene sets of GSEA (Subramanian et al., 2005).

### GST Pull-Down Assay, Western Blot, and Hematoxylin–Eosin Staining

GST-tagged segments of the five domains have been indicated in Figure 2A; GST-tagged Tex13a and empty vector encoding GST (pGEX4t-1) were expressed, respectively, in BL21(DE3) induced by 0.5 mM IPTG for 2 h. GST-tagged proteins were isolated by Glutathione Sepharose 4B beads (GE Health). His6-HA-tagged full-length Tex13a was similarly expressed in BL21(DE3), isolated with Ni-NTA Sepharose FF column (GE Health, Piscataway, NJ, United States), and desalted by dialysis. GST-tagged recombinant proteins bound in Glutathione Sepharose 4B beads were mixed with His6-HA-tagged Tex13a in the mild lysis buffer [50 mM Tris (pH 7.4), 150 mM NaCl, 0.1% TRITON X-100] with proteinase inhibitor cocktail on ice for 3 h. Following washing, the different bound proteins on Glutathione Sepharose 4B beads were diluted with reduced glutathione and were subjected to SDS-PAGE electrophoresis. SDS-PAGE gels were either transferred to PVDF membrane for Western blots analysis or stained by Coomassie brilliant blue. GST-tagged Tex13a beads were incubated for 3 h on ice with testis homogenates diluted in the abovementioned mild lysis buffer. The bound proteins were subjected to Western blotting.

For Western blot analysis, 40-μg protein samples were subjected to SDS-PAGE, and then transferred to PVDF membranes. After blocking with skimmed milk, the membrane was incubated with primary antibodies anti-HA (1:2,000, KM8004, Sungene Biotech, Tianjin, China) and anti-Cnot1 (1:500, 14276-1-AP, Proteintech, Wuhan, China) overnight at 4°C. The membrane was then incubated with the following secondary antibodies for 30 min: goat anti-rabbit IgG H&L (Alexa Fluor 790, Abcam) and goat anti-mouse IgG H&L (Alexa Fluor 791, Abcam). The membranes were thereafter detected on an Odyssey infrared imaging system (LI-COR Biosciences, Lincoln, NE, United States).

Formalin-fixed paraffin-embedded mouse testis sections of 5 μm thickness were used for HE staining.

### Sperm Motility Assay

Male mice aged 3–10 months were used to extract sperm from the caudal epididymis. HS solution containing 25 mM NaHCO_3_ was used to dilute and release the sperm for motility detection. Sperm samples were injected into 80-μm-deep chambers (Hamilton-Throne, Beverly, MA, United States) and analyzed on a computer-assisted sperm analysis (CASA) system (Hamilton-Throne, Beverly, MA, United States) on a Zeiss microscope with a 10× objective (Oberkochen, German). Statistical analysis was performed *via* the unpaired *t*-test incorporated in GraphPad Prism 8. The sperm samples from 14 KO mice and 13 WT mice were analyzed.

#### Data Availability

The raw scRNA-Seq data and disposed data have been deposited in the GEO database (GSE183626).

## Results

### Tex13a-KO Led to a Delayed Degradation of mRNAs Encoding Structural Components of Elongating Spermatids

*Tex13a* is an X-linked gene with two exons in the mouse, both of which were knocked out by CRISPR/Cas9-mediated genome editing (Supplementary Figure 1A). In line with an earlier report (Lu et al., 2019), Tex13a-KO mice were fertile and no visible phenotype was found upon examining of the testis slices stained by HE (Supplementary Figure 1B).

**FIGURE 1:**
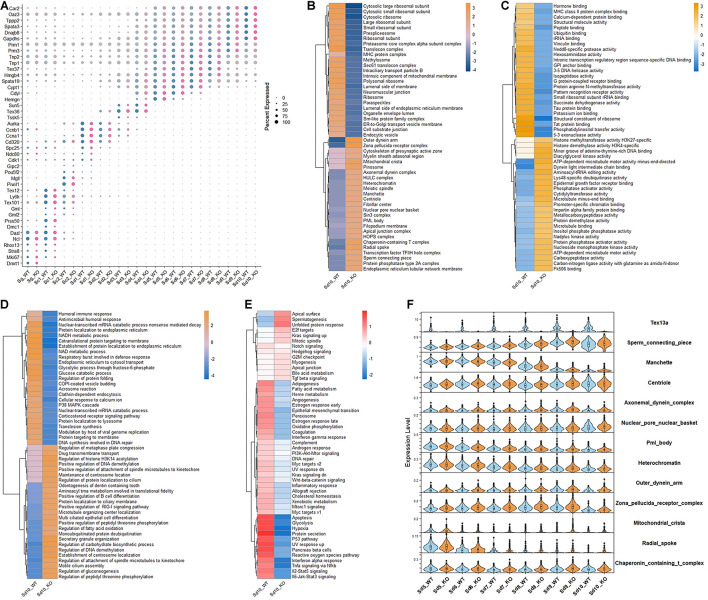
The altered gene expression patterns in the elongating spermatids in Tex13-KO and WT mouse. **(A)** The cell markers of all the germ cell clusters in WT and Tex13a KO mouse testes. **(B)** Top 25 upregulated and downregulated pathways of C5 (CC) (GSEA) in elongating spermatid Sd10 from WT and Tex13a KO mouse testes. **(C)** Top 25 upregulated and downregulated pathways of C5 (MF) (GSEA) in elongating spermatid Sd10. **(D)** Top 25 upregulated and downregulated pathways of C5 (BP) (GSEA) in Sd10_WT and Sd10_KO. **(E)** The relative expression level of 50 hallmark signaling pathways (GSEA) in Sd10_WT and Sd10_KO. **(F)** The expression profiles in Sd5–Sd10 of selected upregulated pathways in Sd10_KO.

To further delineate the function of *Tex13a*, scRNA-seq was performed in wild-type (WT) and *Tex13a* knocked-out (KO) mice. One cluster of spermatogonia, two clusters of spermatocytes, one mixed cell cluster of spermatocyte and early spermatids (Sd1), and nine clusters of haploid spermatids were divided (Supplementary Figure 2A). The well-known cell markers of each cluster were plotted in Figure 1A. Moreover, by examining the differentially expressed genes (DEGs) during the middle and late steps of spermatids (Sd5-Sd10) in between WT and KO, we found that Sd10_WT and Sd10_KO had more significant DEGs, which were enriched in proteasomal proteins, catabolic process, and protein translation pathways (Supplementary Figures 2B–D).

CAMERA enrichment analysis was performed for comparing the overall changes in each gene set or signaling pathway between Tex13a-KO and WT mice (Wu and Smyth, 2012; Cillo et al., 2020). CAMERA enrichment analysis of C5 (CC) pathway gene sets (GSEA) revealed that the gene sets involved in the structural components of sperm tail and head (sperm connecting piece, manchette, centriole, axonemal dynein complex, nuclear pore nuclear basket, outer dynein arm, zona pellucida receptor complex, mitochondrial crista, radial spoke, nuclear pore nuclear basket, PML body, heterochromatin, and chaperonin-containing T complex) were significantly upregulated. Meanwhile, components of the ribosomes were downregulated in the last detectable step of ESs (Sd10) by scRNA-seq (Figure 1B). CAMERA enrichment analysis of C5 (MF) and C5 (BP) pathway gene sets (GSEA) further indicated the existence of extensive alterations of the transcriptome in Sd10 ESs (Figures 1C,D). Furthermore, a 50 hallmark gene sets enrichment analysis revealed that the pathways of various house-keeping genes were globally lower in Sd10_KO than in Sd10_WT, potentially due to competition between junk mRNAs and house-keeping gene transcripts (Figure 1E), which will be discussed further later. Most of the differentially expressed gene sets emerged in Sd10 and some were also found in Sd9, which coincided with the highest expression level of Tex13a in Sd9 and Sd10 (Figure 1F). Therefore, Tex13a could be involved in the regulation of overall RNA metabolism during the late ESs.

### Tex13a Was Revealed to Be a Testis-Specific Component of the CCR4–NOT Complex

**FIGURE 2:**
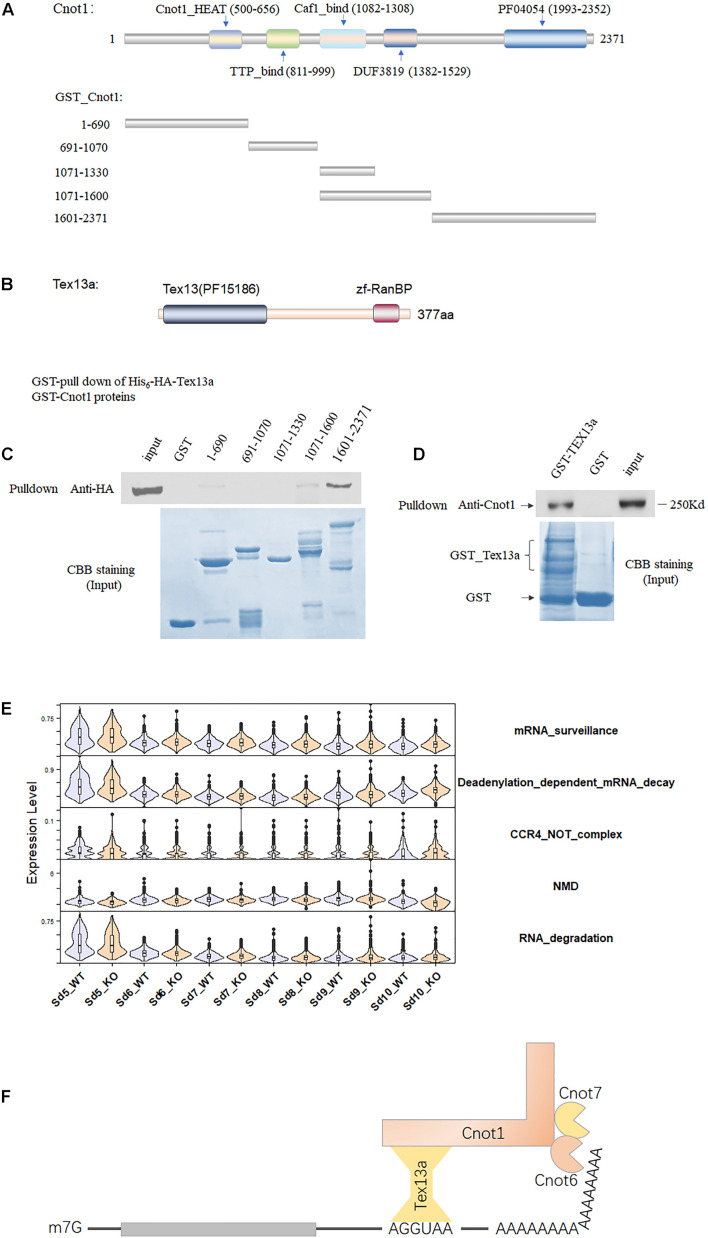
The association of Tex13a with the CCR4–NOT complex. **(A)** The five domains of Cnot1, the five truncated segments of Cnot1 in fusion with GST in the N-terminus facilitated recombinant protein expression. **(B)** Diagram showing the domains of Tex13a. **(C)** GST pull-down analysis of His6-HA-Tex13a with the five recombinant GST-tagged segments of Cnot1 *in vitro*. Recombinant proteins were stained with Coomassie brilliant blue (CBB) staining. **(D)** Endogenous Cnot1 of mouse testes was pulled down by recombinant GST_Tex13a. **(E)** The expression profiles of four RNA metabolism-related pathways from Sd5 to Sd10 in between WT and KO. **(F)** A proposed interaction model of Tex13a with CCR4–NOT complex.

To further verify the involvement of Tex13a in the RNA metabolism, we analyzed the potential association of Tex13a with the CCR4–NOT complex, which serves as a key RNA machine participating in the initial step of RNA degradation. Among the several important components of the CCR4–NOT complex, Cnot1 was the largest and main structural component with five domains (Chalabi Hagkarim and Grand, 2020). The five domains annotated by Pfam have been shown in Figure 2A. Tex13a was difficult to be detected by Western blot or immunofluorescence for heterologous expression of HA-tagged Tex13a in cell lines (data not shown). As a result, the full-length Tex13a in fusion with His6-HA tags at the N-terminus and the five recombinant segments in fusion with GST corresponding to the five domains were constructed, expressed, and extracted from *E. coli* for GST pull-down analysis (Figures 2A,B). As expected, GST pull-down showed a direct interaction of Tex13a with Cnot1. The interaction was found to be mediated by the C-terminal HEAT domain (pfam domain PF04054) of Cnot1 (Figure 2C). In turn, GST-Tex13a also pulled down endogenous Cnot1 from the mouse testis homogenates (Figure 2D). As a feedback effect, Tex13a deletion led to the upregulation of deadenylation-dependent mRNA decay and CCR4–NOT pathways, but without changing the level of the RNA degradation pathway (Figure 2E). We proposed a novel interacting model of Tex13a and CCR4–NOT complex based on the consensus AGGUAA motif bound by Tex13a and the interaction of Tex13a with Cnot1, which gave the CCR4–NOT complex the potential to cause targeted mRNA degradation in late steps of ESs (Figure 2F).

### Tex13a Deletion Altered the Sperm Motility

**FIGURE 3:**
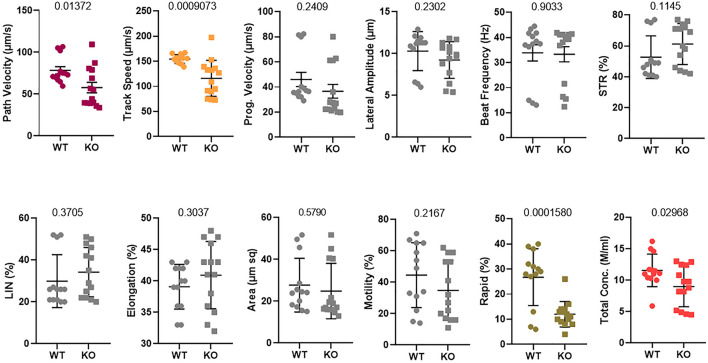
Sperm motility was analyzed using a computer-assisted sperm analysis (CASA) system. Twelve different parameters were assayed and compared between WT and Tex13a_KO mice. *p*-value was displayed above each image.

The delayed degradation of RNAs of sperm structural components did not result in a visible phenotype in the morphology of the mature sperm and fertility (Lu et al., 2019). Additionally, it remarkably altered the different parameters of sperm motility, such as path velocity (VAP, average path velocity) (*p* = 0.01372), track speed (VCL, velocity curvilinear) (*p* = 0.0009073), and rapid progression (*p* = 0.0001580) as well as the sperm total concentration (*p* = 0.02968) (Figure 3). However, the beat frequency was not changed. Therefore, the rapid motility was significantly altered in Tex13-KO mouse sperm. Hence, a significant reduction of rapid motility could be considered as a typical phenotype marker of *Tex13a* deletion.

## Discussion

Various mRNAs can accumulate during the late stages of ESs, causing delayed translation. These junk RNAs should be eliminated as soon as their tasks are completed. This was demonstrated by an abrupt degradation of mRNAs in Sd10 and a transitory upregulation of CCR4–NOT pathway genes in the late steps of ESs (Figure 2E). Chromatid bodies have been regarded as an important center for the RNA metabolic process *via* regulating NMD in spermatids (MacDonald, 2019). NMD is involved in the surveillance and degradation of aberrant RNAs *via* affecting NMD pathways, though it is also capable of degrading a subset of functional intact RNAs (He and Jacobson, 2015; Jones and Wilkinson, 2017). Deadenylation, the rate-limiting step of mRNA degradation, initiates all major mRNA decay pathways identified so far in eukaryotes (Chen and Shyu, 2011). Thereafter, following shortening of the poly(A) tail, the mRNAs can be degraded in a 3′–5′ direction by the exosomes, or, alternatively, deadenylation is followed by decapping (a backup way) and degradation in a 5′–3′ direction (Parker and Song, 2004; Houseley and Tollervey, 2009). Mammalian mRNA deadenylation involves two consecutive phases mediated by the PAN2-PAN3 for long poly(A) and the CCR4–NOT complexes for short poly(A) (Bartlam and Yamamoto, 2010; Wahle and Winkler, 2013). Therefore, degradation of the intact mRNAs during the late steps of ESs were likely initiated by deadenylation, and NMD might not serve as a major degradation pathway, which was also evidenced by the decreased level of NMD in Sd10 (Figure 2F). The C-terminal module of CNOT1 contains a rigid scaffold consisting of two perpendicular stacks of HEAT-like repeats (Boland et al., 2013). Moreover, based on the interacting details of Tex13a with Cnot1 C-terminus, the binding model of Cnot2 and Cnot3 with Cnot1 C-terminus can provide a useful clue (Boland et al., 2013), but the application of crystallography might be required in the future investigations.

As to the converse co-relationship between spermatid-specific gene levels and house-keeping genes in late steps of ESs (Figures 1B,E), a possible explanation could be that the timely degradation of junk RNAs possibly made space for low-level house-keeping genes due to the limited spaces for storage. “Promiscuous” transcription has been reported to exist in haploid spermatids; an array of genes were found to be transcribed extremely high while the other genes were transcribed at lower levels (Schmidt and Schibler, 1995; White-Cooper and Davidson, 2011). Such low-level transcripts were possibly more vulnerable to be downregulated by delayed degradation of junk mRNAs during spermiogenesis. Most of such genes have been derived from the general pathways involved in the various house-keeping roles (Figure 1E). However, more detailed information about the exact RNA motif and mRNAs targeted by Tex13a *in vivo* are needed in the future. For example, identification of new approaches regulating RNA–protein interactions as CLIP can be used for monitoring the RNA motifs bound by Tex13 family members (Nechay and Kleiner, 2020; Masuda et al., 2021).

In terms of the role of Tex13a in the mRNA turnover, only sperm motility alteration might not significantly affect the fertility when Tex13a was deleted, because no alternation of litter size and fertility was recorded (Lu et al., 2019). This is in line with the previous studies that have indicated that only sperm motility is less predictive for evaluating male fertility (Broekhuijse et al., 2012; Wang and Swerdloff, 2014; Boe-Hansen and Satake, 2019). Additionally, the detailed information about parameters of the percentage of spermatozoa with the cytoplasmic droplets (distal cytoplasmic droplets and proximal cytoplasmic droplets), sperm head (length, width, perimeter, and area), midpiece and tail (length and abnormalities), acrosome integrity, plasma membrane integrity, mitochondria activity, DNA damage, histones, chromatin defects, ROS levels, calcium channel activity, pH, and acrosome reaction can also be helpful in complete phenotyping of Tex13a_KO in future studies. The genes derived from the skeletal structures, energy metabolism, mitochondria, and calcium channels may affect the sperm dynamics more directly. Furthermore, animal pairing experiments might also be worth establishing to test the sperm penetration rate *in vitro*, pregnancy rate, farrowing rate and litter size. In this report, we only displayed the changes of sperm motility parameters to verify the potential role of Tex13a in the turnover of mRNAs associated with the different structural components of sperm, many of which were directly related to sperm motility parameters. Screening of differentially expressed genes in protein level related to sperm motility should also be carried out in future studies.

It also needs to be highlighted that the weak phenotype in the spermiogenesis in Tex13a-KO mice may be compensated by other members of the *Tex13* family (Lu et al., 2019). Therefore, Tex13c, which was expressed in late steps of spermatids, also should be paid more attention (Kim et al., 2021).

In summary, *Tex13a* deletion resulted in a delayed degradation of mRNAs of spermiogenesis-related structural components, which potentially resulted in the reduction of house-keeping genes. Sperm motility was also affected, especially the rapid motility was significantly reduced. Interestingly, Tex13a was able to bind Cnot1, a core component of the CCR4–NOT complex, which may affect the decay of particular junk mRNAs during the late steps of ESs.

## Data Availability Statement

The datasets presented in this study can be found in online repositories. The names of the repository/repositories and accession number(s) can be found in the article/Supplementary Material.

## Ethics Statement

The animal study was reviewed and approved by University of Nantong Animal Care and Use Committee and the Animal Care and Use Office.

## Author Contributions

FS, CC, YT, and WQ performed supervision, funding acquisition, project administration, and methodology. PM performed sperm motility, HE staining, and genotyping. XC performed DNA cloning, recombinant protein isolation, GST pull-down, and Western blot. JW and XL provided technical assistance, data curation, and project supervision. JC and YH performed animal model preparation. YL performed data analysis, designed experiment, and wrote the manuscript. All authors contributed to the article and approved the final manuscript.

## Conflict of Interest

The authors declare that the research was conducted in the absence of any commercial or financial relationships that could be construed as a potential conflict of interest.

## Publisher’s Note

All claims expressed in this article are solely those of the authors and do not necessarily represent those of their affiliated organizations, or those of the publisher, the editors and the reviewers. Any product that may be evaluated in this article, or claim that may be made by its manufacturer, is not guaranteed or endorsed by the publisher.
