# Supplementary material for: Tex13a Optimizes Sperm Motility via Its Potential Roles in mRNA Turnover
Source: Front Cell Dev Biol. 2021 Oct 18;9:761627. doi: 10.3389/fcell.2021.761627 (PMC8558480; doi:10.3389/fcell.2021.761627)
Supplement: Supplementary file 1 [file Data_Sheet_1.docx]

**Tex13a optimizes sperm motility via its potential roles in mRNA turnover**

Yinchuan Li, Panpan Mi, Xue Chen, Jiabao Wu, Xiaohua Liu, Yunge Tang, Jinmei Cheng, Weibing Qin, C. Yan Cheng, Fei Sun

**Supplementary Figures**


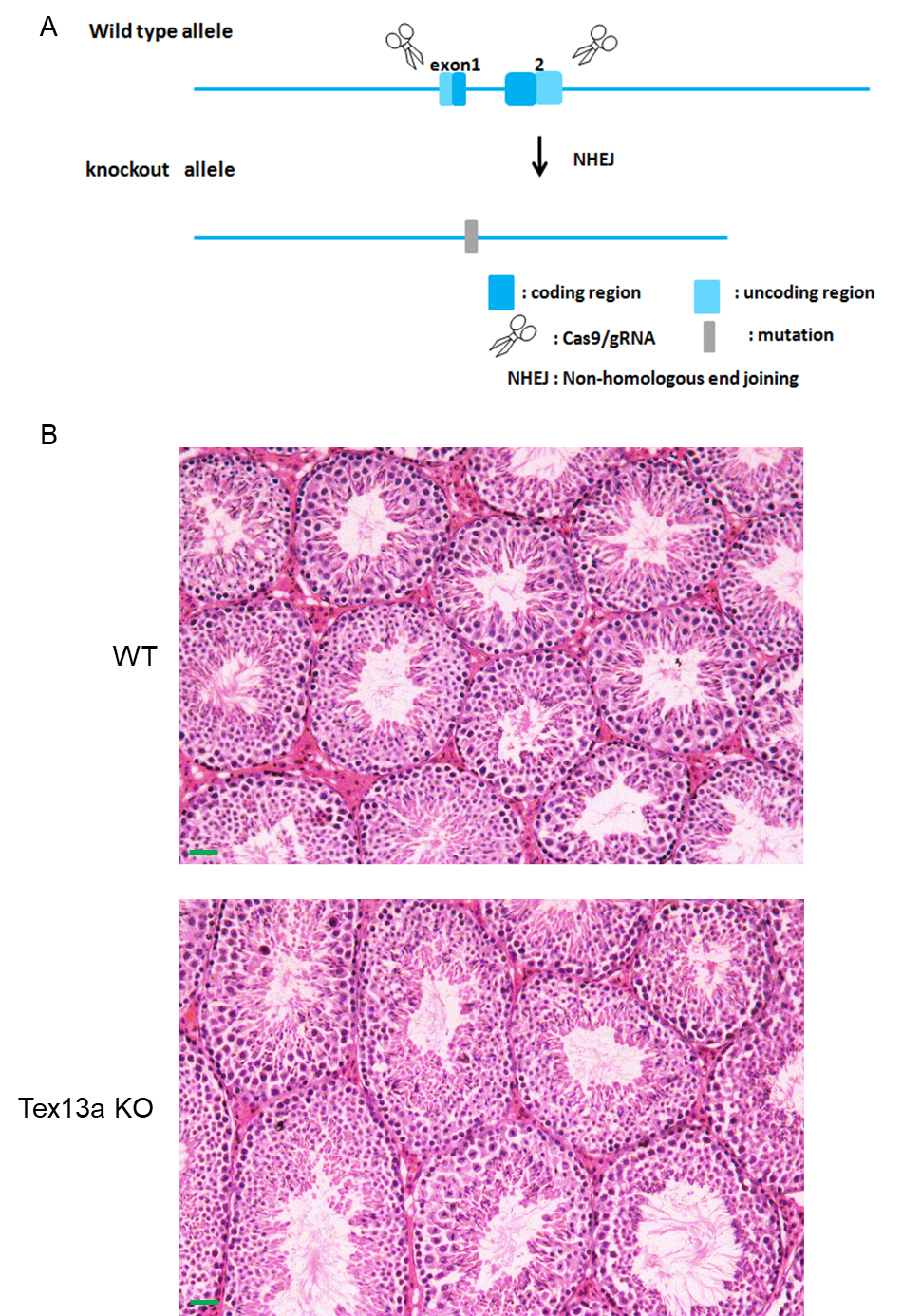


Figure S1. Tex13a-KO and phenotype. (A), Diagram showing the WT allele and KO allele of *Tex13a* in mouse by Crispr-Cas9. (B), HE staining of the testes slices of WT and Tex13a KO mice. Bar, 50 μm.


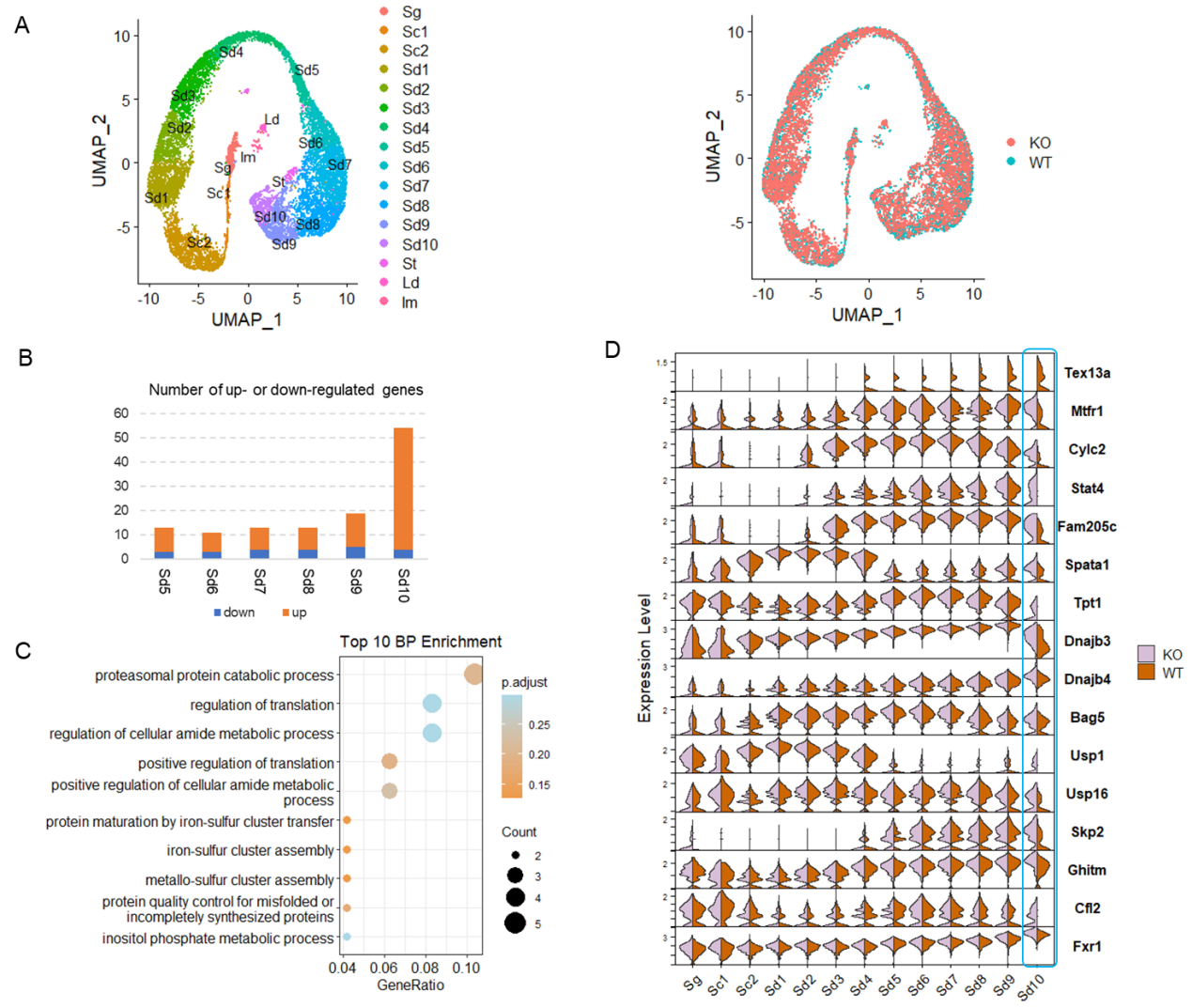


Figure S2. Differentially expressed genes (DEGs) in the elongating spermatids between Sd10_KO and Sd10_WT. (A), UMAP plot of the cells of testes from WT and Tex13a KO mice split by cell clusters (left panel) or by samples (right panel). (B), DEGs were generated by FindMarkers in R package Seurat (test.use = "bimod", logfc.threshold = 0.1). Up-regulated genes (p≤0.05, p_val_adj≤0.05, logFC≥0.2, PCT1≥ 0.2) and down-regulated genes (p≤0.05, p_val_adj≤0.05, logFC≤-0.2, PCT2≥ 0.2) were filtered as significant DEGs in between WT and KO. (C), The top 10 GO (BP) enrichment analysis of the significant DEGs in Sd10, generated by R package clusterProfiler. (D), The violin plot of selected DEGs across all the germ cell clusters in WT and KO samples. Genes in Sd10 were boxed.
